# Novel Pandemic Influenza A(H1N1) Viruses Are Potently Inhibited by DAS181, a Sialidase Fusion Protein

**DOI:** 10.1371/journal.pone.0007788

**Published:** 2009-11-06

**Authors:** Gallen B. Triana-Baltzer, Larisa V. Gubareva, John M. Nicholls, Melissa B. Pearce, Vasiliy P. Mishin, Jessica A. Belser, Li-Mei Chen, Renee W. Y. Chan, Michael C. W. Chan, Maria Hedlund, Jeffrey L. Larson, Ronald B. Moss, Jacqueline M. Katz, Terrence M. Tumpey, Fang Fang

**Affiliations:** 1 NexBio, Inc., San Diego, California, United States of America; 2 Influenza Division, National Center for Immunization and Respiratory Diseases, Centers for Disease Control and Prevention, Atlanta, Georgia, United States of America; 3 Departments of Pathology and Microbiology, University of Hong Kong, Pok Fu Lam, Hong Kong Special Administrative Region, People's Republic of China; University of California San Francisco, United States of America

## Abstract

**Background:**

The recent emergence of a novel pandemic influenza A(H1N1) strain in humans exemplifies the rapid and unpredictable nature of influenza virus evolution and the need for effective therapeutics and vaccines to control such outbreaks. However, resistance to antivirals can be a formidable problem as evidenced by the currently widespread oseltamivir- and adamantane-resistant seasonal influenza A viruses (IFV). Additional antiviral approaches with novel mechanisms of action are needed to combat novel and resistant influenza strains. DAS181 (Fludase™) is a sialidase fusion protein in early clinical development with *in vitro* and *in vivo* preclinical activity against a variety of seasonal influenza strains and highly pathogenic avian influenza strains (A/H5N1). Here, we use *in vitro*, *ex vivo*, and *in vivo* models to evaluate the activity of DAS181 against several pandemic influenza A(H1N1) viruses.

**Methods and Findings:**

The activity of DAS181 against several pandemic influenza A(H1N1) virus isolates was examined in MDCK cells, differentiated primary human respiratory tract culture, *ex-vivo* human bronchi tissue and mice. DAS181 efficiently inhibited viral replication in each of these models and against all tested pandemic influenza A(H1N1) strains. DAS181 treatment also protected mice from pandemic influenza A(H1N1)-induced pathogenesis. Furthermore, DAS181 antiviral activity against pandemic influenza A(H1N1) strains was comparable to that observed against seasonal influenza virus including the H274Y oseltamivir-resistant influenza virus.

**Conclusions:**

The sialidase fusion protein DAS181 exhibits potent inhibitory activity against pandemic influenza A(H1N1) viruses. As inhibition was also observed with oseltamivir-resistant IFV (H274Y), DAS181 may be active against the antigenically novel pandemic influenza A(H1N1) virus should it acquire the H274Y mutation. Based on these and previous results demonstrating DAS181 broad-spectrum anti-IFV activity, DAS181 represents a potential therapeutic agent for prevention and treatment of infections by both emerging and seasonal strains of IFV.

## Introduction

In the United States alone influenza virus (IFV) causes over 200,000 hospitalizations annually and is responsible for approximately 36,000 deaths every year [1], [2]. The emergence of antigenically novel strains or drug-resistant IFV strains is of major public health concern in light of the burden of seasonal influenza and the persistent threat of pandemic influenza. Indeed, the recent outbreak of pandemic influenza A(H1N1) (novel 2009 A(H1N1), “swine influenza”) is a reminder that non-human IFV are capable of evolving to infect humans, and as such remain a continuous public health threat [3]–[6]. Antigenically novel strains such as pandemic IFV A(H1N1) present a particular problem as the human population is largely serologically naïve to the novel virus, and vaccination with seasonal influenza vaccine does not elicit protective levels of cross-reactive antibody to the novel virus [7]. In addition, antiviral resistance among certain influenza strains is a growing problem [8]–[11]. Nowhere is this more evident than in the dramatic rise of oseltamivir-resistant seasonal H1N1 influenza and adamantane-resistant seasonal H3N2 viruses in the past few years [8], [10], [12]–[14]. Predicting the future evolution of influenza viruses and their sensitivity to available antivirals and vaccines remains an ongoing public health concern. Additional effective anti-influenza therapeutics are clearly needed [15].

The pandemic influenza A(H1N1) outbreak is believed to have originated in central Mexico in the spring of 2009 and rapidly spread across the globe. This strain of IFV contains gene segments from common avian, swine and human influenza strains [16]. While birds are a well known reservoir of a great variety of antigenically distinct influenza subtypes (e.g., highly virulent H5N1), swine are considered the “mixing vessel” for generating novel strains of IFV that can also infect humans [6]. This “mixing vessel” effect is due to the fact besides the classic swine viruses, pigs are susceptible to infection by both avian and human influenza viruses. Co-infection with viruses of various origins allows for reassortment of gene segments and a genesis of viruses with a new gene constellation such as seen in triple reassortment swine viruses [6]. Even with this apparent mechanism for rapid evolution in place most influenza viruses of avian or swine origin have exhibited difficulty in transmission between humans [6], [17], [18]. In contrast one of the alarming hallmarks of the pandemic influenza A(H1N1) virus is that it appears readily transmissible between humans.

Because new IFV strains often are so antigenically different from seasonal human IFV, current vaccines and preexisting serum antibodies are unlikely to offer effective protection; indeed this is the case with the novel 2009 pandemic influenza A(H1N1) [7]. This fact, and the time lag involved in producing a pandemic influenza A(H1N1) vaccine, suggests that acute approaches such as antiviral drugs are needed to complement the available infection control measures.

DAS181 (Fludase™) is a recombinant fusion protein composed of the catalytic domain of *Actinomyces viscosus* sialidase and the epithelial anchoring domain of human amphiregulin (AR). This 45 kDa protein has been shown to bind to cells and efficiently remove cell-surface sialic acid residues from respiratory epithelium [19], [20]. Sialic acid is the primary receptor for IFV binding and entry into the host cell, therefore removal of sialic acid by DAS181 potently inhibits IFV infection [19], [21]. Furthermore, by targeting the host cells rather than the virus, DAS181 may be less likely to induce drug resistance than virus-targeting compounds (e.g. adamantanes and neuraminidase inhibitors). DAS181 has demonstrated inhibition of infection by over 30 IFV strains in MDCK cells, human airway epithelium cultures (wdHAE), *ex vivo* human lung and bronchi tissues, mice, and ferrets [19], [20], [22]–[24]. Furthermore, DAS181 also protected mice from lethal challenge with highly pathogenic avian influenza virus (HPAI) (H5N1) with treatment as late as 72 hours post-infection [22].

Because of the broad-spectrum activity of DAS181 against both laboratory and clinically isolated IFV strains, we hypothesized that it might also be effective against the novel pandemic influenza A(H1N1) virus. Here we show potent *in vitro*, *ex vivo*, and *in vivo* inhibition of several 2009 pandemic influenza A(H1N1) clinical isolates and an oseltamivir-resistant clinical isolate, by DAS181.

## Results

### DAS181 Inhibits Pandemic Influenza A(H1N1) Clinical Isolates In Vitro

**Figure 1:**
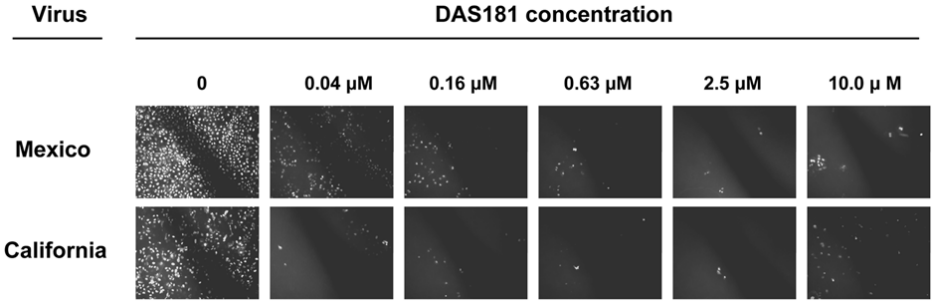
DAS181 inhibits pandemic IFV A(H1N1): Immunofluorescent detection of 2009 A(H1N1) foci in MDCK cells. MDCK cells were pretreated with DAS181 for 2 hours (at indicated concentrations) before infection with either pandemic influenza A(H1N1) A/Mexico/4604/09 (Mexico) or A/California/04/09 (California). After 24 hrs post-infection, the cultures were fixed and processed for immunostaining to detect virus antigen as marker of infected cells. White spots indicate infected cells that form foci (clusters) of different size ranging from 5 to 100's of individual infected cells.

To determine if DAS181 is active against the pandemic influenza A (H1N1) (2009 A(H1N1)) two viruses obtained from Mexico and California in April of 2009 were tested for sensitivity to DAS181. MDCK cells pretreated with DAS181 (2 hrs) were inoculated with these viruses and subsequent viral replication was compared to that in untreated MDCK cells at 24 hrs post-infection. To this end, cell culture supernatants were harvested to determine the infectious virus yields (infectious progeny production/release) while the MDCK cell monolayers were inspected for the presence of infected cells forming foci (clusters of infected cells) using immunofluorescent detection of the viral NP antigen. Numerous and large sized foci were detected in untreated MDCK cell culture whereas in DAS181 pre-treated monolayers, the foci were very small in size and a qualitative dose-dependent reduction in foci number was also observed. DAS181 concentrations as low as 0.04 µM substantially reduced the amount of virus-positive foci using the A/California/4/2009 or A/Mexico/4604/2009 H1N1 viruses (Figure 1). Quantifying the number of infected foci indicated DAS181 EC50 values to be <0.04 µM (Table 1). Quantifying the amount of virus progeny in the cell supernatants also revealed consistent results and a dose-dependent reduction of pandemic IFV A(H1N1) replication by DAS181 (Table 2). In comparison, DAS181 was similarly effective against two seasonal influenza A/H1N1 strains (one oseltamivir-sensitive and one oseltamivir-resistant) in the same assay (Table 1, Table 2). This result shows that DAS181 is highly active against the pandemic IFV A(H1N1) in MDCK cells, and suggests the level of activity is comparable to that against seasonal IFV A.

**Table 1:** DAS181 inhibits pandemic IFV A(H1N1): Number of Viral Foci in MDCK culture (per well).

Virus	PBS	DAS181 0.04 µM	DAS181 0.16 µM	DAS181 0.63 µM	DAS181 2.5 µM	DAS181 10 µM
A/Mexico/4604/2009	80±9	1±0	0	0	0	0
A/California/04/2009	180±15	3±1	1±0	0	0	0
A/Hawaii/31/2007	20±6	5±1	2±1	0	0	0
A/Hawaii/21/2007 (H274Y)	26±4	0	0	0	0	0

MDCK cells were pretreated with DAS181 for 2 hours (at indicated concentrations) before infection with A/Mexico/4604/2009, A/California/04/2009, or seasonal influenzas A/Hawaii/31/2007 or A/Hawaii/21/2007 (oseltamivir-resistant H274Y mutant) at M.O.I. ranging from 0.001 to 0.005 MOI. At 24 hrs p.i. the cells were immunostained to detect NP viral antigen and the number of positive foci per individual well was counted (“Number of Viral Foci”). Simultaneously the viral yield in the culture supernatants (media above the cell monolayers) collected from individual well was determined (Table 2). Values represent mean±SD of quadruplicate experiments.

**Table 2:** DAS181 inhibits pandemic IFV A(H1N1): Viral Yield in MDCK culture (log10 infectious particles per 100 µl).

Virus	PBS	DAS181 0.04 µM	DAS181 0.16 µM	DAS181 0.63 µM	DAS181 2.5 µM	DAS181 10 µM
A/Mexico/4604/2009	5.4±0.4	4.4±0.4	<1	<1	<1	<1
A/California/04/2009	3.5±02	3.1±0.3	2.8±0.7	2.0±0.7	<1	<1
A/Hawaii/31/2007	5.4±0.4	3.2±1.1	2.5±1.2	<1	<1	<1
A/Hawaii/21/2007 (H274Y)	5.3±0.3	<1	2.3±2.4	<1	<1	<1

MDCK cells were pretreated with DAS181 for 2 hours (at indicated concentrations) before infection with A/Mexico/4604/2009, A/California/04/2009, or seasonal influenzas A/Hawaii/31/2007 or A/Hawaii/21/2007 (oseltamivir-resistant H274Y mutant) at M.O.I. ranging from 0.001 to 0.005 MOI. At 24 hrs p.i. the cells were immunostained to detect NP viral antigen (Table 1). Simultaneously the viral yield in the culture supernatants (media above the cell monolayers) collected from individual well was determined and expressed as log10 infectious particles per well (“Viral Yield”), a measure of released infectious viral progeny. Limit of detection in viral yield assay is 10 infectious particles per 100 µl and all values below this are marked as <1. Values represent mean±SD of quadruplicate experiments.

**Figure 2:**
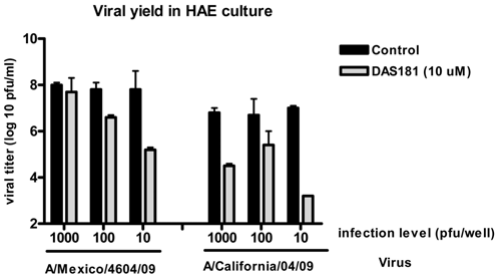
DAS181 inhibits pandemic IFV A(H1N1): viral yield in 2009 A(H1N1) infected HAE culture. Well differentiated primary human airway epithelium cultures (HAE) were pretreated with DAS181 for 2 hours (at 10 µM) before infection with either A/Mexico/4604/2009 or A/California/04/2009 at indicated infection levels. After 24 hrs the apical wash was collected and viral yield was determined on MDCK cells as described in Materials and Methods. Values represent mean±SD of quadruplicate analysis of each sample.

The A/Mexico/4604/2009 and A/California/04/2009 A(H1N1) isolates were also tested for DAS181 sensitivity in a well differentiated primary human airway epithelium culture model (HAE). The HAE model better represents the human airway (compared to cell line models) in that it contains all the functional cell types found in the airway (ciliated, secretory, and basal cells) and more accurately represents the expression pattern of sialic acids found in the upper human respiratory tract [25]–[30]. The HAE model supported robust infection by both 2009 A(H1N1) viruses and DAS181 strongly inhibited replication of both strains at several multiplicities of infection (Figure 2).

### DAS181 Inhibits Pandemic Influenza A(H1N1) Virus in Ex Vivo Human Bronchi Tissue

**Figure 3:**
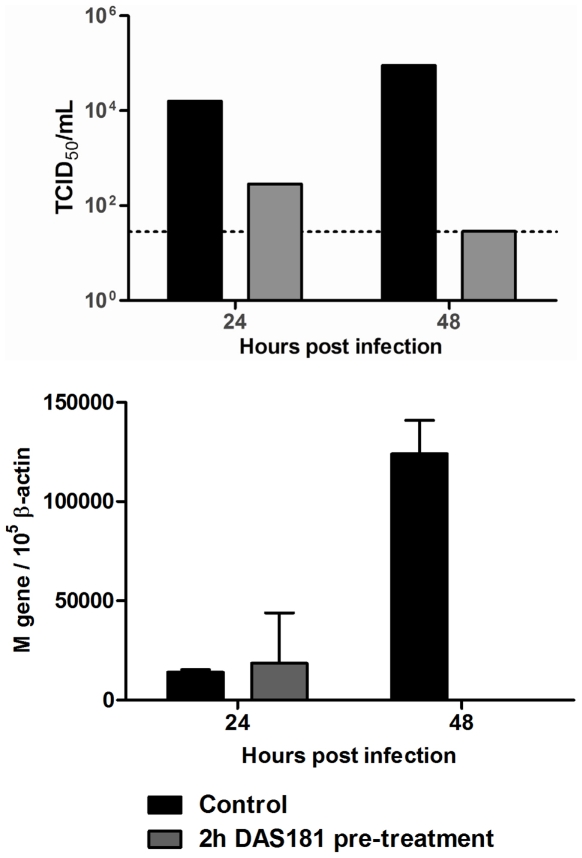
DAS181 inhibits pandemic IFV A(H1N1) viral replication in *ex vivo* human bronchi tissue. Human bronchi tissues were collected from patients undergoing lung resection and immediately placed on air-liquid interface membranes for culturing. Within 12 hours the tissues were pretreated with PBS (“control”) or DAS181 (10 µg/cm^2^, “2 h DAS181 pretreatment”) for 2 hours at 37°C. After washing, the tissues were infected with pandemic influenza A(H1N1) A/California/04/2009 (10^6^ TCID_50_/tissue) for 1 hr and then washed. Tissues were then incubated at 37°C for 24 or 48 hrs before washing the apical surface and determining viral titer in either the apical wash by TCID_50_ on MDCK cells (top panel), or within tissue homogenate by RT-PCR analysis of viral M-gene RNA level normalized to cellular β-actin RNA level (bottom panel). Dotted line represents limit of detection of the TCID_50_ assay.

Because MDCK cells do not accurately represent the human respiratory tract in terms of cell type and fine sialic acid structure milieu, and HAE cultures are extensively cultured *in vitro*, the activity of DAS181 against pandemic IFV A(H1N1) was tested using fresh human bronchi tissue. Tissues were obtained from patients undergoing lung resection and immediately placed on sponge membranes for short term culturing. Bronchi tissue infected with the pandemic IFV A(H1N1) A/California/04/2009 replicated the virus to high titer as analyzed at 24 and 48 hrs post-infection. Bronchi tissue pretreated with DAS181 (2 hours at 10 µg/cm^2^) revealed dramatically less viral replication at both time points, as determined by TCID_50_ analysis of infectious viral yield, with total inhibition at 48 hrs. Analysis of total viral yield in tissue homogenates, by qRT-PCR analysis of viral M-gene RNA level, corroborated the pandemic IFV A(H1N1) inhibition in bronchi by DAS181 (Figure 3).

### DAS181 Inhibits Pandemic Influenza A(H1N1) Clinical Isolates in Mice

**Figure 4:**
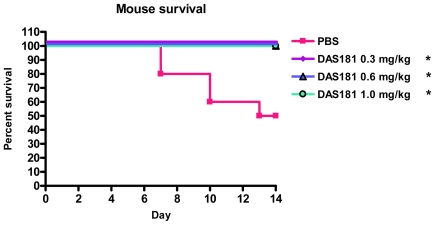
DAS181 inhibits pandemic IFV A(H1N1)-induced lethality in mice. BALB/c mice were inoculated with the pandemic influenza A(H1N1) virus A/Mexico/4108/2009 (1000 MID_50_/mouse, *i.n.*). The mice were treated with PBS or DAS181 (0.3, 0.6, or 1 mg/kg) *q.d.x5*, beginning 6 hours post-infection. Survival was tracked daily for 14 days. Values represent percent survival amongst 10 mice per group. All DAS181 treated groups had 100% survival. Statistical significance from PBS determined by Kaplan-Meier Log Rank Test, * = p<.05.

**Figure 5:**
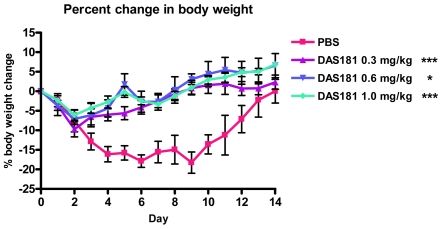
DAS181 inhibits pandemic IFV A(H1N1)-induced body weight loss in mice. BALB/c mice were inoculated with the pandemic influenza A(H1N1) virus A/Mexico/4108/2009 (1000 MID_50_/mouse, *i.n.*). The mice were treated with PBS or DAS181 (0.3, 0.6, or 1 mg/kg) *q.d.x5*, beginning 6 hours post-infection. Body weight was measured daily for 14 days p.i., and is expressed normalized to starting body weight. The data is collected from the same animals/study shown in Figure 4. Values represent mean±SEM body weight change amongst 10 mice per group. Statistical significance from PBS determined by analysis of Area Under Curve (AUC) with ANOVA and Bonferroni post-test, * = p<0.05, *** = P<0.001.

**Figure 6:**
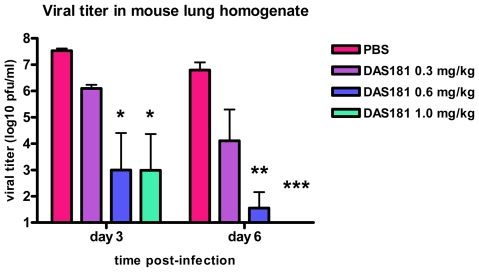
DAS181 inhibits pandemic IFV A(H1N1) viral replication in mice. BALB/c mice were inoculated with the pandemic influenza A(H1N1) virus A/Mexico/4108/2009 (1000 MID_50_/mouse, *i.n.*). The mice were treated with PBS or DAS181 (0.3, 0.6, or 1 mg/kg) *q.d.x5*, beginning 6 hours post-infection. Lungs were harvested on day 3 and day 6 p.i.. Viral titer in lung homogenate was determined by standard plaque assay on MDCK cells. Values represent mean±SEM viral titer amongst 3 mice per group/day. PFU = plaque forming units. No virus was detectable on day 6 in the DAS181 1.0 mg/kg treatment group. Statistical significance from PBS treatment determined by ANOVA with Bonferroni post-test, * = p<0.05, ** = p<0.01, *** = p<0.001. The limit of detection is 100 PFU/ml.

To better understand the affect of DAS181 on pandemic IFV A(H1N1) infection in a mammalian animal model, the A/Mexico/4108/2009 virus used to infect BALB/c mice in a manner yielding significant lethality, body weight loss, and viral replication in the lungs. Intranasal DAS181 treatment was performed daily for 5 days beginning 6 hours post-infection at several dose levels (0.3, 0.6, or 1 mg/kg/day). DAS181 treatment completely protected against pandemic IFV A(H1N1)-induced lethality (Figure 4) and reduced pandemic IFV A(H1N1)-induced body weight loss, particularly at later time points in infection (>day 2) (Figure 5). DAS181 treatment also reduced pandemic influenza A(H1N1) viral replication in a dose-dependent manner, as measured by viral titer in the lungs of the mice at day 3 and day 6 post-infection (Figure 6). These results indicate that DAS181 effectively inhibits novel pandemic influenza A(H1N1) virus replication and morbidity in mice.

## Discussion

The current outbreak of pandemic influenza A(H1N1) that was first detected in Mexico is a reminder that IFV is an evolving pathogen capable of significant reassortment events resulting in the generation of novel strains. The “Spanish flu” pandemic of 1918–1919 infected approximately 20% of the world's population and killed 20–50 million people, making it the most devastating disease in all recorded history [31], [32]. The initial epidemiology of the pandemic influenza A(H1N1) virus suggests a lower case fatality rate compared to the “Spanish flu”, however concerns remain with regard to the potential for secondary waves of infection, as occurred with the “Spanish flu”, resulting in higher mortality compared with the initial wave of infection [5].

Amidst a ∼99% frequency of oseltamivir resistance in 2008–2009 seasonal A/H1N1 influenza isolates the novel 2009 pandemic influenza A(H1N1) was fortuitously found to retain oseltamivir sensitivity. However, this virus acquired the adamantane resistance-conferring gene via a reassortment event [16], [33], [34]. Co-circulation of viruses with different drug resistance profiles in humans poses a threat of emergence of viruses resistant to both oseltamivir and adamantanes [35]. Simultaneous infection of a single host with both strains could lead to gene reassortment and ultimate production of a virus with both oseltamivir-resistance and the characteristics of the novel influenza A(H1N1) virus. The process of swapping gene segments between two viral strains (segment reassortment, genetic drift) and has been widely reported to be a major mechanism of viral evolution [36], [37]. Because the pandemic influenza A(H1N1) virus, or any other emerging strain of influenza, could potentially gain the oseltamivir-resistance mutation (H274Y) it is critical to continue to develop anti-influenza compounds with alternative mechanisms of action.

DAS181 is a sialidase fusion protein currently in development for use as a broad-spectrum inhibitor of influenza virus and parainfluenza virus infection. DAS181 acts by reducing viral binding to the respiratory epithelium, a novel mechanism of action for anti-influenza drugs, and as such it complements existing anti-influenza approaches. Furthermore, since DAS181 targets the host cell, the potential for generating viral resistance may be less than with traditional influenza inhibitors, which target the virus itself (M2 inhibitors, neuraminidase inhibitors). Here we demonstrate for the first time that DAS181 inhibits replication of several pandemic influenza A(H1N1) viruses in MDCK cells, HAE culture, *ex vivo* human bronchi tissue and in a murine model. The effective inhibitory DAS181 concentration for the pandemic influenza A(H1N1) viruses was similar to that with seasonal IFV, suggesting that the pandemic influenza A(H1N1) viruses binds to sialic acid residues comparably recognized by DAS181. This finding highlights the potential broad spectrum activity of DAS181 against current and future IFV strains.

A common hallmark of novel IFV strains is altered sialic acid (Sia) recognition. All influenza A viruses bind to terminal Sia acid residues expressed on the cellular receptors. However, the efficiency of this interaction depends on several factors such as the structure of adjacent oligosaccharides. When avian or swine viruses cross the inter-species barrier and infect humans they may interact differently with the ‘human’ Sia-containing receptors, as evidenced by HPAI H5N1 and 1918 “Spanish flu” viruses isolated from clinical material [18], [38], [39]. While the exact Sia residues recognized by these emerging strains (pandemic influenza A(H1N1) or HPAI) may vary, DAS181 exhibits broad spectrum sialic acid removal and therefore may be effective against current and future influenza viruses with altered HA-Sia binding specificity.

The data obtained using MDCK cell culture should be interpreted with caution because this commonly used cell line may not accurately represent the human respiratory tract in terms of cell types and expression pattern of various sialic acids [26], [27]. Furthermore, the mouse infection data is limited by the fact that the mouse and human respiratory systems present different viral binding patterns [40], [41], and the novel 2009 A(H1N1) virus is not a mouse-adapted strain. As such the dynamics of 2009 A(H1N1) infection shown in these models may not accurately represent infection in humans. It should be noted that in the absence of daily ketamine anesthesia, A/Mexico/4108/2009 does not result in substantial morbidity or mortality in the mouse model. However, the significant protective efficacy of DAS181 under these experimental conditions is remarkable and the further evaluation of DAS181 utilizing additional A(H1N1) viruses is warranted. In contrast, the DAS181 effectiveness shown here in HAE culture and *ex vivo* bronchi model, two of the most representative models of the human respiratory tract, correlates with the MDCK and mouse data and suggests that DAS181 may indeed be effective against pandemic influenza A(H1N1) infections although further clinical confirmation is required. Additionally, it will be valuable to evaluate the affect of treatment timing relative to infection; in the studies herein treatment was initiated within 6 hours of infection. We have previously observed that NAIs, oseltamivir and zanamivir, do not inhibit DAS181 activity *in vitro* or *in vivo* (unpublished data). In cell culture, DAS181 exhibits a strongly synergistic relationship with the NAIs against a panel of laboratory IFV strains (unpublished data), although this has not been examined in the context of 2009 A(H1N1) infection.

Because of the potential for emerging IFV strains to attain oseltamivir resistance via reassortment with the current seasonal H1N1 strain we also tested DAS181 activity against an oseltamivir-resistant IFV. The most common mutation conferring oseltamivir-resistance involves H274Y (H275Y in N1 numbering) substitution in the neuraminidase of viruses of N1 antigenic subtype (H1N1 and H5N1). This mutation was originally identified in isolates from oseltamivir-treated individuals infected with H1N1 virus [42], and more recently, in patients infected with HPAI of H5N1 background, a troubling observation given the pandemic potential and extremely virulent nature of this IFV strain [43]–[45]. Perhaps more alarming was the finding that seasonal H1N1 viruses, carrying the oseltamivir resistance-conferring H274 mutation, emerged simultaneously in several countries in 2007–2008, including countries where oseltamivir is not prescribed [10], [12]–[14]. The frequency of IFV isolates with this mutation reached ∼99.5% in the US in 2008–2009 [34]. Here we show that influenza A/Hawaii/21/2007, one of the first oseltamivir-resistant viruses from the 2007–2008 season, is highly sensitive to DAS181 in MDCK cells. This *in vitro* finding indicates that DAS181 may be active against currently circulating oseltamivir-resistant IFV and further suggests that if novel strains, such as the pandemic influenza A(H1N1) viruses, attain the H274Y mutation, DAS181 may be active against these new isolates as well. Additional testing of DAS181 sensitivity of several NAI-resistant viruses is ongoing.

While the 2009 pandemic IFV A(H1N1) and the oseltamivir-resistant seasonal IFV of 2007–2009 currently exhibit low case fatality rates, these patterns are potentially unstable. Given the IFV' propensity for rapid evolution, the constant threat of an emerging highly virulent drug-resistant strain is concerning and supports the need to monitor evolution of the 2009 A(H1N1) IFV strains and simultaneously develop specific vaccines and novel antivirals.

The safety and efficacy of DAS181 as a novel anti-IFV agent is currently being evaluated in clinical trials. Based on its broad spectrum preclinical *in vitro* and *in vivo* activity against seasonal and potentially pandemic IFV, as well as against the 2009 pandemic IFV A(H1N1) and oseltamivir-resistant IFV, DAS181 represents a potentially valuable treatment option for emerging and drug-resistant IFV.

## Materials and Methods

### Cells and Viruses

Madin Darby Canine Kidney (MDCK) cells were obtained from American Type Culture Collection (Manassas, VA). Growth and differentiation of primary human bronchial epithelial cells to produce HAE culture were performed as described previously [46], [47]. Briefly, primary human bronchial cells (Cell Applications, San Diego, CA) expanded to passage 3 were seeded in porous membrane inserts (4.5 µm pore size, 12 mm diameter, Corning, Corning, NY) at the density of 5×10^4^ cells/cm^2^. Three days after seeding the cells, the medium from the apical side was removed and the confluent monolayers were cultured at an air-liquid interface. The medium from the basal compartment was replaced daily, and the *in vitro* differentiation of primary cells was achieved after 4–6 weeks.

The seasonal A/Hawaii/31/2007 (H1N1) and closely related A/Hawaii/21/2007 (H274Y) (H1N1) virus isolates, and the 2009 pandemic A(H1N1) virus isolates A/California/04/2009, A/Mexico/4604/2009, and A/Mexico/4108/2009 were obtained from Dr. Alexander Klimov, Influenza Division, Centers for Disease Control and Prevention, Atlanta, Georgia, USA. The A/California/04/2009, A/Mexico/4604/2009 viruses were propagated in MDCK cells. The A/Mexico/4108/09 virus was isolated from nasopharyngeal swabs collected from a patient with severe respiratory illness and subsequently propagated in embryonated hen's eggs as previously described [22].

### Determination of Virus Titer/Yield in Cell Culture Supernatant by Immunofluorescence

Confluent MDCK cell monolayers in 96-well plates were washed with PBS and infected with 10-fold virus dilutions (culture media from treated/infected MDCKs or apical washes from treated/infected HAE, see below). After 1 hour adsorption at 4°C, unbound virus was removed and cells were overlaid by fresh DMEM containing 0.2% BSA and 2 µg/ml TPCK-treated trypsin. Plates were incubated at 37°C for 24 hours, at which point, the supernatants were discarded and cells were fixed with ice cold methanol/acetic acid (v/v 95/5). Fixed cell monolayers were immunostained with primary anti-influenza A nucleoprotein (NP) mouse monoclonal antibody (kind gift of Dr. Robert Webster, St. Jude Children's Research Hospital, Memphis, TN) and FITC-conjugated secondary antibody (Sigma, St. Louis, MO). Cell clusters (foci) expressing viral antigen NP were counted using a fluorescent microscope (Axiovert 200, Zeiss, Germany) and viral titers were calculated. An individual focus is formed by a cluster of at least 5 neighboring virus infected cells, although a much greater number of individual cells (∼100) was observed in untreated infected cells.

### Testing DAS181 Sensitivity Using MDCK Infection

Confluent MDCK cells in 96-well plate format were pretreated with DAS181 for 2 hours at 37°C (diluted in EDB-BSA buffer, 50 uL/well) before washing twice with PBS. MDCK cells were then infected with the influenza strains at multiplicity (MOI) ranging from 0.001 to 0.005. After 1 hr adsorption at 4°C, cells were washed twice with PBS and replenished with fresh DMEM media (containing 0.2% BSA and 2 µg/ml TPCK-treated trypsin). Cultures were incubated at 37°C for 24 hrs at which point the antiviral activity of DAS181 was determined in each well by two criteria: a number of infectious centers (immunofluorescent foci) (photo Fig. 1, Table 1) and viral yield in cell media (Table 1). Virus yield in supernatants was determined as described above. Each drug dilution was tested in quadruplicate and viral yield vales are expressed as mean±standard deviation. The MDCK experiments were performed twice, yielding comparable results.

### Testing DAS181 Activity Using HAE Infection

HAE cultures in 12-well plate format were pretreated with DAS181 for 2 hours at 37°C (diluted in EDB-BSA buffer, 100 uL/well) before washing with PBS and infection with the novel 2009 A(H1N1) viruses. The cultures were infected with 1000, 100, or 10 infectious units per insert for 1 hr at 4°C before washing with PBS. Cultures were incubated at 37°C for 24 hrs before washing the apical surface of the culture with 200 ul PBS and storing the collected wash at −80°C for quantitative determination of viral titer as described above. The HAE experiment was performed once.

### Ex Vivo Human Bronchi Tissue Infection and Treatment

Fresh bronchi tissue was removed from patients undergoing lung resection and immediately sectioned into multiple biopsies of approximately 3×5×5 mm dimensions. After sectioning, the tissues were immediately placed on sponge membranes with culture medium (F-12K nutrient mixture with L-glutamine, and antibiotics) contacting the basal portion of the tissues (establish air-liquid interface), before infection and/or DAS181 treatment within 12 hours of resection. The tissues were treated with PBS or DAS181 (10 ug/cm^2^) for 2 hours at 37°C on the apical surface before washing with PBS and then infecting with A/California/04/09 for 1 hr at 37°C as described previously [48]. After washing off unbound virus the tissues were incubated at 37°C for 24 or 48 hrs. At each time point the apical surface of the tissues was washed with PBS and the viral titer in the apical wash was determined by TCID_50_ analyses on MDCK cells. The *ex vivo* bronchi experiment was performed twice (individual tissues/patients), yielding comparable results. Each experiment was analyzed by TCID_50_ and qRT-PCR.

### qRT-PCR Analysis of Viral M-Gene RNA Level

Analysis of total viral yield in bronchi tissue homogenate was performed as described previously [49]. In brief, total nucleic acid was extracted from the bronchi tissue using NucliSens easyMAG extraction system (bioMerieux, Netherlands) according to manufacturer's instructions. 12 µl of extracted nucleic acid was used to prepare cDNA by Invitrogen Superscript III kit with random primer as described previously [50]. 2 µl of cDNA was amplified in LightCycler with a total volume of 20 µl reaction containing FastStart DNA Master SYBR Green I Mix reagent kit (Roche Diagnostics GmbH, Germany), 4.0 mM MgCl2 and 0.5 mM of each primer. The forward primer (5′-CTTCTAACCGAGGTCGAAACG-3′) and the reverse primer (5′-GGCATTTTGGACAAAKCGTCTA-3) were used for amplification corresponding to the M gene of influenza A [51]. All M-gene quantities are expressed normalized to B-actin level. Cycling conditions were as follows: an initial denaturation at 95°C for 10 minutes, followed by 40 cycles of 95°C for 10 seconds, 60°C for 3 seconds, 72°C for 12 seconds with ramp rates of 20°C/second. A series of dilutions were prepared to generate calibration curves and run in parallel with the test samples. At the end of the assay, PCR products were subjected to a melting curve analysis to determine the specificity of the assay.

### Mouse Infection and Treatment

In a pilot experiment (not shown) the mouse 50% infectious dose (MID_50_) was determined for the pandemic IFV A/Mexico/4108/09 (∼32 PFU/mouse). Female BALB/c mice, 10 to 11 weeks old, (The Jackson Laboratory, Bar Harbor, MI) were randomly divided into 4 groups (16 mice/group). On day 0 all mice were intranasally inoculated with 50 µL of A/Mexico/4108/09 (1000 MID_50_/mouse = ∼32,000 PFU/mouse). All animals were anesthetized via ketamine (100 mg/kg) and held in an upright position when receiving the nasal drops. At 6 hours post-inoculation all animals received the intranasal treatment of either DAS181 or vehicle control (PBS) in 50 µL volume. Group 1 served as control and received PBS, and groups 2–4 received DAS181 at 0.3, 0.6, or 1 ml/kg, respectively. Treatments were repeated daily for a total of 5 treatments (*q.d.x5*). Ten mice per group were observed daily for morbidity, as measured by weight loss, or mortality, for 14 days post-infection (p.i.) Body weight is represented as percent change from day 0 body weight (immediately prior to experiment). For viral titer analysis, whole lungs were collected from euthanized animals on day 3 and day 6 p.i. (3 mice/group/day). Lung tissues were collected and titrated for virus as described previously [52]. The mouse experiment was performed twice with comparable results. All animal research was conducted under the guidance of CDC's Institutional Animal care and Use Committee and in an Association for Assessment and Accreditation of Laboratory Animal Care International- accredited facility.

### Viral Titer Determination by Plaque Assay

Viral replication in mouse lung homogenate was quantified on MDCK cells to determine infectious titer (plaque forming units per mL, pfu/ml). In brief 6×10-fold serial dilution was performed on the lung homogenate samples followed by 1 hr binding at 37°C on confluent MDCK cells in 6-well plate format. After washing off unbound virus with PBS, the cells were overlaid with 1∶1 Noble Agar (1.8%) and 2x DME-F12 (supplemented with Glutamax (Invitrogen, Carlsbad, CA), ITS (Invitrogen), and 3 µg/ml acetylated trypsin (Sigma, St. Louis, MO)). After allowing agar to solidify, the plates were incubated for ∼48 hrs at 37°C before fixing with ethanol and staining with crystal violet and counting plaque number at each dilution.

### Data Analysis

All data was graphed with Prism 4.02 software and significance was determined by ANOVA with Bonferroni post-test, except for mouse survival studies which utilized Kaplan-Meier Log Rank test.
